# Supplementary material for: Clonal origin and development of high hyperdiploidy in childhood acute lymphoblastic leukaemia
Source: Nat Commun. 2023 Mar 25;14:1658. doi: 10.1038/s41467-023-37356-5 (PMC10039905; doi:10.1038/s41467-023-37356-5)
Supplement: Supplementary file 1 — Supplementary Figures and Tables [file 41467_2023_37356_MOESM1_ESM.pdf]

SUPPLEMENTARY INFORMATION FOR

Clonal origin and development of high hyperdiploidy in childhood acute lymphoblastic leukemia

Eleanor L Woodward[1], Minjun Yang[1], Larissa H Moura-Castro[1], Hilda van den Bos[2], Rebeqa Gunnarsson[1], Linda Olsson-Arvidsson[1,3], Diana CJ Spierings[2], Anders Castor[4], Nicolas Duployez[5,6], Marketa Zaliova[7,8], Jan Zuna[7,8], Bertil Johansson[1,3], Floris Foijer[2], Kajsa Paulsson[1]

[1]Department of Laboratory Medicine, Division of Clinical Genetics, Lund University, Lund, Sweden, [2]European Research Institute for the Biology of Ageing (ERIBA), University of Groningen, University Medical Center Groningen, Groningen, The Netherlands, [3]Department of Clinical Genetics, Pathology, and Molecular Diagnostics, Office for Medical Services, Region Skåne, Lund, Sweden, [4]Department of Pediatrics, Skåne University Hospital, Lund University, Lund, Sweden, [5]Laboratory of Hematology, Centre Hospitalier Universitaire (CHU) Lille, Lille, France, [6]Unité Mixte de Recherche en Santé (UMR-S) 1172, INSERM/University of Lille, Lille, France, [7]Department of Pediatric Hematology and Oncology, Second Faculty of Medicine, Charles University/University Hospital Motol, Prague, Czech Republic, [8]Childhood Leukaemia Investigation Prague (CLIP), Prague, Czech Republic.

Supplementary Table 1. Patient and genetic data for nine cases of high hyperdiploid childhood acute lymphoblastic leukemias subjected to single cell whole genome sequencing

Case	Gender	Karyotype[a]	No. of somatic mutations	Genes targeted by non-silent somatic mutations	Other somatic changes
1	M	56,XY,+X,+Y,dup(1)(q21q32),+4,+6,+10,+14,+17,+18,+21,+21	1,045	*CCDC63, MARVELD2, MRAP2, NPAS3, NRAS, OR1D5, SCN4A, TLN2*	
2	M	56,XY,+X,dup(1)(q21q41),+4,+6,+8,+10,+14,+17,+18,+21,+21	1,005	*AC012593.1, BEND5, FLT3, GIF, PCDHGA5, RBFOX1, RP11-347L18.1, SLC7A4, SULF1*	Focal *ETV6* deletion
3	M	66,XY,+X,dup(1)(q21q44)x2,+3,+4,+5,+6,+8,+9,+10,+10,+11,+12, 13,del(13)(q21q31),+14,+14,+16,+16,del(16)(p12)x2,+17,+18,+18, +21,+21	1,043	*ATP8A2, CNTN1, HYDIN, NAV3, NOC2L, POLR3E, ROBO2, SPATA31D1, TCP10, TCTN3, TEKT4, TSEN34, UGT8*	
4	F	57,XX,+X,+X,der(3)t(3;6)(q29;q22),+4,+8,+10,+14,+del(14)(q12),+17, +18,+21,+21	1,108	*CDKN2A, DPP10, GPR4, HSPG2, HUWE1, IKZF1, LL22NC01-81G9.3, PCDH15, TBR1, TEX28, ZSCAN16*	
5	M	57,XY,+X,+del(5)(q23),+6,del(7)(q34),+10,+dup(14)(q11q22), +del(16)(p13),+17,+18,+21,+21,+mar	1,857	*ADAM12, AKAP6, COL4A6, CREBBP, DSCAML1, FLT3, HSD3B2, KLHL36, LAMA1, MAOA, MAPK4, MUC16, MUC17, MYPN, OTOA, PCSK6, RNF130, STON1-GTF2A1L, SULT1B1, TEP1, TGM2, TRRAP, TUBA4A, UBA6, ZNF804A*	UPID11, UPID13, subclonal focal *ETV6* deletion
6	M	54,XY,+X,+4,+6,+9,+10,+18,+21,+21	837	*ACOT1, DOT1L, FLT3, KRAS, SIGLEC1, STOX2, TENM3*	
7	F	54,XX,+X,+4,+6,+14,+17,+18,+21,+21	1,632	*ADH7, AR, ATF7IP, DHCR7, FAM27E1, FAM27E3, GATA5, GRIK2, HYDIN, KRAS, NRAS, OR56A1, PADI1, PTPN11, TBPL2, WDHD1*	Focal *PAX5* deletion
8	M	51,XY,+X,+4,+14,+21,+21	822	*CILP, F11, FAM81A, FLT3, GATA1, HSF5, KCNH7, MATN3, PTK2*	
9	M	56,XY,+X,+4,+4,+8,+14,+17,+18,+21c,+21,+21	1,734	*APOL3, CAMLG, CREBBP, CSMD3, CTD-2128A3.2, DNTT, FN1, IFT122, KRAS, LPHN3, LRP3, LUC7L, M1AP, MYB, PRICKLE2, RP11-763F8.1, SGOL1, SMARCD1, THSD4, TNPO2, ZAR1L*	

[a]Based on G-banding and SNP array analysis on bulk DNA

Abbreviations: F, female; M, male; UPID, uniparental isodisomy

Supplementary Table 2. Subclonality involving whole chromosomes in 577 cases of high hyperdiploid pediatric acute lymphoblastic leukemia

Chromosome	No of cases with subclonality (%)	Type of subclonality (No of cases)										*P* disomy vs UPID[a]
		Trisomy/ disomy	Trisomy/ UPID	Trisomy/ tetrasomy 2:2	UPID/ disomy	Disomy/ tetrasomy 2:2	Disomy/ monosomy	Tetrasomy 2:2/ pentasomy	Trisomy/ tetrasomy 3:1	XXY/XX0	XY/X0	
1	2 (0.35)	1	0	0	1	0	0	0	0	0	0	
2	3 (0.52)	3	0	0	0	0	0	0	0	0	0	0.593
3	5 (0.87)	5	0	0	0	0	0	0	0	0	0	0.263
4	9 (1.6)	7	2	0	0	0	0	0	0	0	0	0.754
5	4 (0.69)	3	1	0	0	0	0	0	0	0	0	1
6	10 (1.7)	9	1	0	0	0	0	0	0	0	0	0.208
7	6 (1.0)	3	3	0	0	0	0	0	0	0	0	0.639
8	26 (4.5)	20	3	2	0	1	0	0	0	0	0	0.0529
9	50 (8.7)	26	22	0	1	0	1	0	0	0	0	0.0969
10	14 (2.4)	9	2	3	0	0	0	0	0	0	0	0.468
11	10 (1.7)	6	2	0	0	1	1	0	0	0	0	0.936
12	5 (0.87)	4	1	0	0	0	0	0	0	0	0	0.922
13	5 (0.87)	2	0	0	0	0	3	0	0	0	0	-
14	8 (1.4)	2	1	5	0	0	0	0	0	0	0	1
15	5 (0.87)	2	3	0	0	0	0	0	0	0	0	0.420
16	7 (1.2)	5	2	0	0	0	0	0	0	0	0	1
17	10 (1.7)	6	3	1	0	0	0	0	0	0	0	1
18	7 (1.2)	0	0	7	0	0	0	0	0	0	0	-
19	0 (0)	0	0	0	0	0	0	0	0	0	0	-
20	1 (0.17)	0	0	0	0	0	1	0	0	0	0	-
21	21 (3.6)	1	0	9	0	0	0	5	6	0	0	-
22	3 (0.52)	2	0	0	0	0	0	0	1	0	0	-
X females	13 (5.1)	1	10	1	0	0	0	1	1	0	0	**0.000260**
X males	0 (0)	0	0	0	0	0	0	0	0	0	0	-
Y	4 (1.2)	0	0	0	0	0	0	0	0	3	1	-
Sum	-	117	56	28	1	2	6	6	8	3	1	0.872

[a]Two-sided exact binomial test for subclonality trisomy/disomy (expected 2/3) vs trisomy/UPID (expected 1/3)

Abbreviations: UPID, uniparental isodisomy

Supplementary Table 3. RMSE values between simulation result and 577 cases of high hyperdiploid pediatric acute lymphoblastic leukemia

UPID frequency at the end of simulation	Modal chromosome number	Comparison group	Trisomy/tetrasomy at different chromosome modal numbers[a]	Trisomy/tetrasomy distribution similarity
2.5%	51-61	Diploid/sequential vs cases	0.81 / 0.37	0.10 / 0.11
		Tetraploid/sequential vs cases	NA / NA [b]	NA / NA [b]
		Diploid/tripolar 3 groups vs cases	0.35 / 0.27	0.087 / 0.035
		Diploid/tripolar 4 groups vs cases	**0.27 / 0.19**	**0.075 / 0.035**
	62-67	Tetraploid/sequential 3 groups vs cases	0.25 / 0.83	0.341 / 0.163
		Tetraploid/sequential 4 groups vs cases	**0.23 / 0.776**	0.339 / 0.166
		Diploid/tripolar 3 groups vs cases	0.61 / 0.94	0.325 / 0.138
		Diploid/tripolar 4 groups vs cases	1.10 / 0.779	**0.271 / 0.128**
5.0%	62-67	Tetraploid/sequential 3 groups vs cases	0.22 / 0.81	0.34 / 0.162
		Tetraploid/sequential 4 groups vs cases	**0.21 / 0.79**	0.339 / 0.166
		Diploid/tripolar 3 groups vs cases	0.47 / 0.81	0.324 / 0.136
		Diploid/tripolar 4 groups vs cases	1.7 / 0.71	**0.275 / 0.13**

[a]The smallest RMSE values are shown in bold

[b]Lack of required virtual cell numbers for RMSE value calculation

Abbreviations: NA, not applicable; RMSE, root mean squared error; UPID, uniparental isodisomy

Supplementary Table 4. Frequency and pattern of structural rearrangements, targeted deletions, and mutations in high hyperdiploid childhood acute lymphoblastic leukemia

**Somatic aberration**	**Total no. (%)**	**No. of clonal (%)**	**No of subclonal (%)**	**Occurred before chromosomal event, no (%)**	**Occurred after chromosomal event, no (%)**
dup(1q)	140/577 (24)	82 (59)	58 (41)	0/8 (0)	8/8 (100)
del(6q)	25/577 (4.3)	15 (60)	10 (40)	0/22 (0)	22/22 (100)
i(7q)	15/577 (2.6)	14 (93)	1 (6.7)	N.I.	N.I.
Partial 17q gain	54/577 (9.4)	38 (70)	16 (30)	N.I.	N.I.
IKZF1 del[a]	23/427 (5.4)	20 (87)	3 (13)	N.I.	N.I.
CDKN2A del	53/427 (12)	42 (79)	11 (21)	1/16 (6.3)	15/16 (94)
PAX5 del	19/427 (4.4)	14 (74)	5 (26)	0/1 (0)	1/1 (100)
ETV6 del	45/427 (11)	27 (60)	18 (40)	1/9 (11)[b]	8/9 (89)
CREBBP del	10/427 (2.3)	8 (80)	2 (20)	0/1 (0)	1/1 (100)
TCF3 del	10/427 (2.3)	9 (90)	1 (10)	N.I.	N.I.
CREBBP mut	21/218 (9.6)	12 (57)	9 (43)	N.I.	N.I.
FLT3 mut	32/218 (15)	15 (47)	17 (53)	0/2 (0)	2/2 (100)
IKZF1 mut	8/218 (3.7)	3 (38)	5 (62)	1/1 (100)	0/1 (0)
KRAS mut	61/218 (28)	31 (51)	30 (49)	0/4 (0)	4/4 (100)
NRAS mut	50/218 (23)	21 (42)	29 (58)	0/1 (0)	1/1 (100)
PTPN11 mut	14/218 (6.4)	7 (50)	7 (50)	0/1 (0)	1/1 (100)
Other mut	155	102 (66)	53 (34)	4/56 (7.1)	52/56 (93)

Abbreviations: N.I., no informative cases; del, deletion; mut, mutation

[a]Includes cases with loss of *IKZF1* through isochromosome 7q and monosomy 7

[b]Constitutional *ETV6* deletion

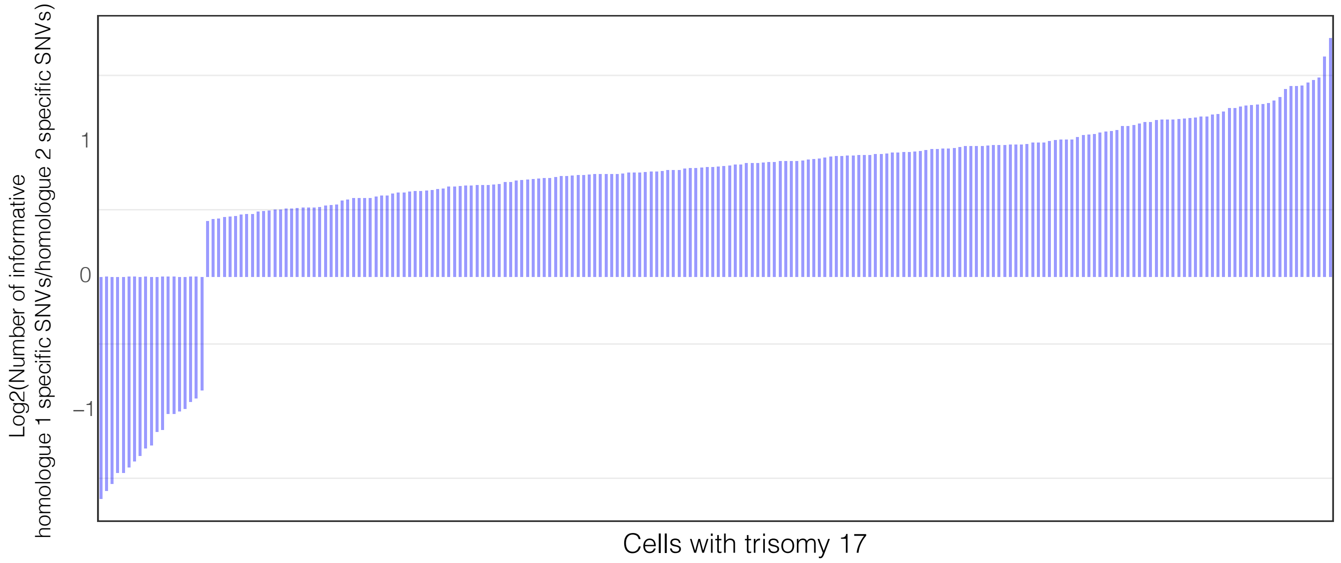

Supplementary Figure 1. Trisomy 17 homologue-specific analysis of scWGS data in case 9, showing gain of homologue 1 in 19 cells and of homologue 2 in 201 cells. Abbreviations: SNV, single nucleotide variant. Source data are provided as a Source Data file.

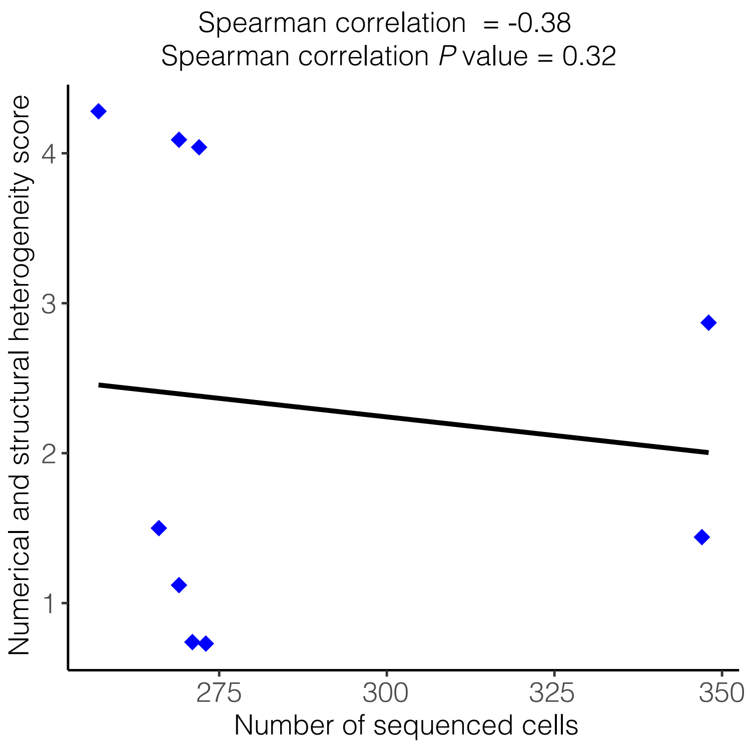

Supplementary Figure 2. Spearman's rank correlation between the number of sequenced cells and the numerical and structural heterogeneity scores. No correlation was seen (*P* = 0.32; Spearman's correlation two-sided test). Source data are provided as a Source Data file.

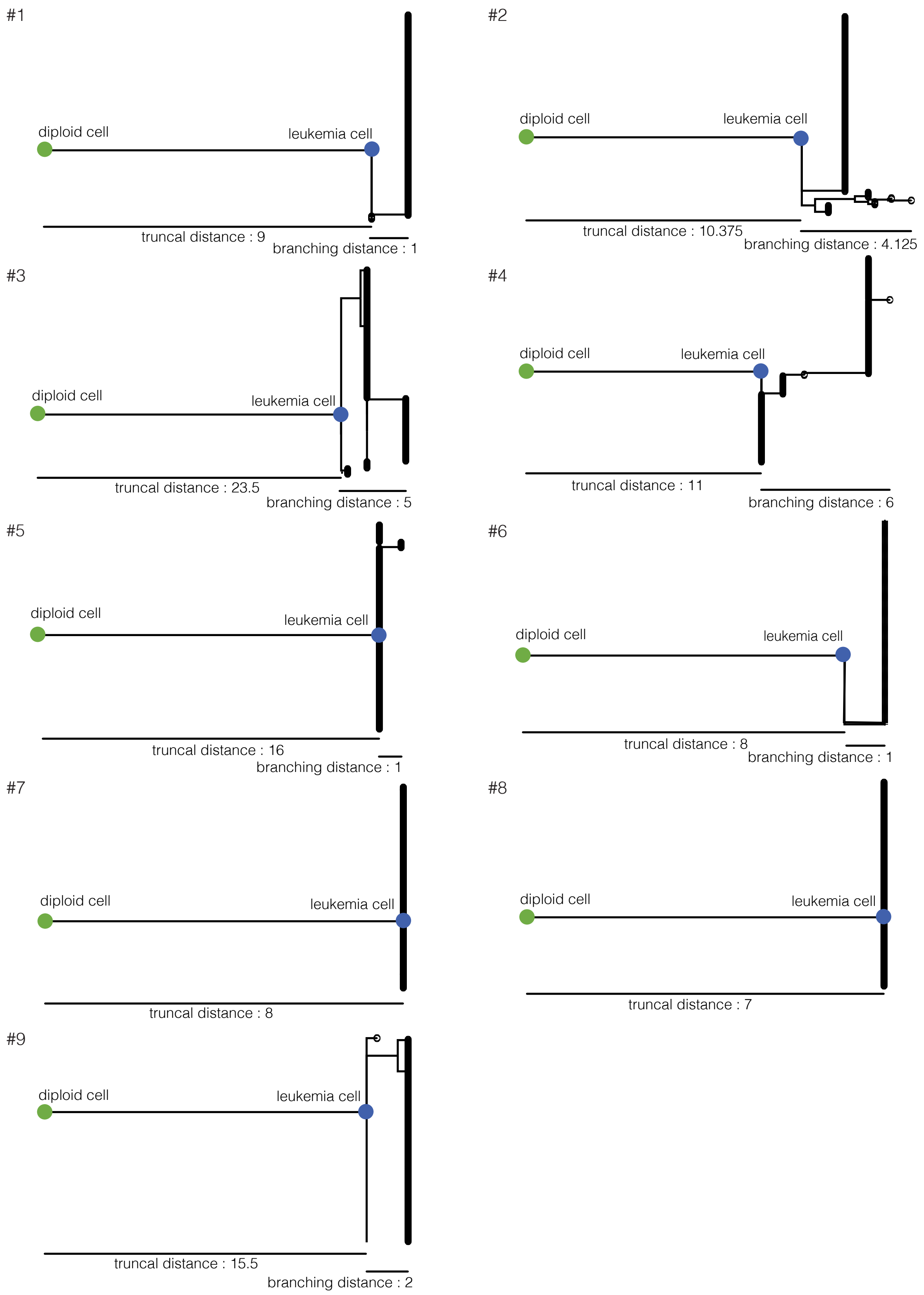

Supplementary Figure 3. Minimum evolution trees of single cell copy number data for nine primary high hyperdiploid childhood acute lymphoblastic leukemia cases. Trees are rooted by simulated normal diploid cells and only the copy number events that were observed in at least two single cells were used. All cases showed relatively long truncal and relatively short branching distances, agreeing with a punctuated evolution model for copy number changes in these malignancies. Source data are provided as a Source Data file.

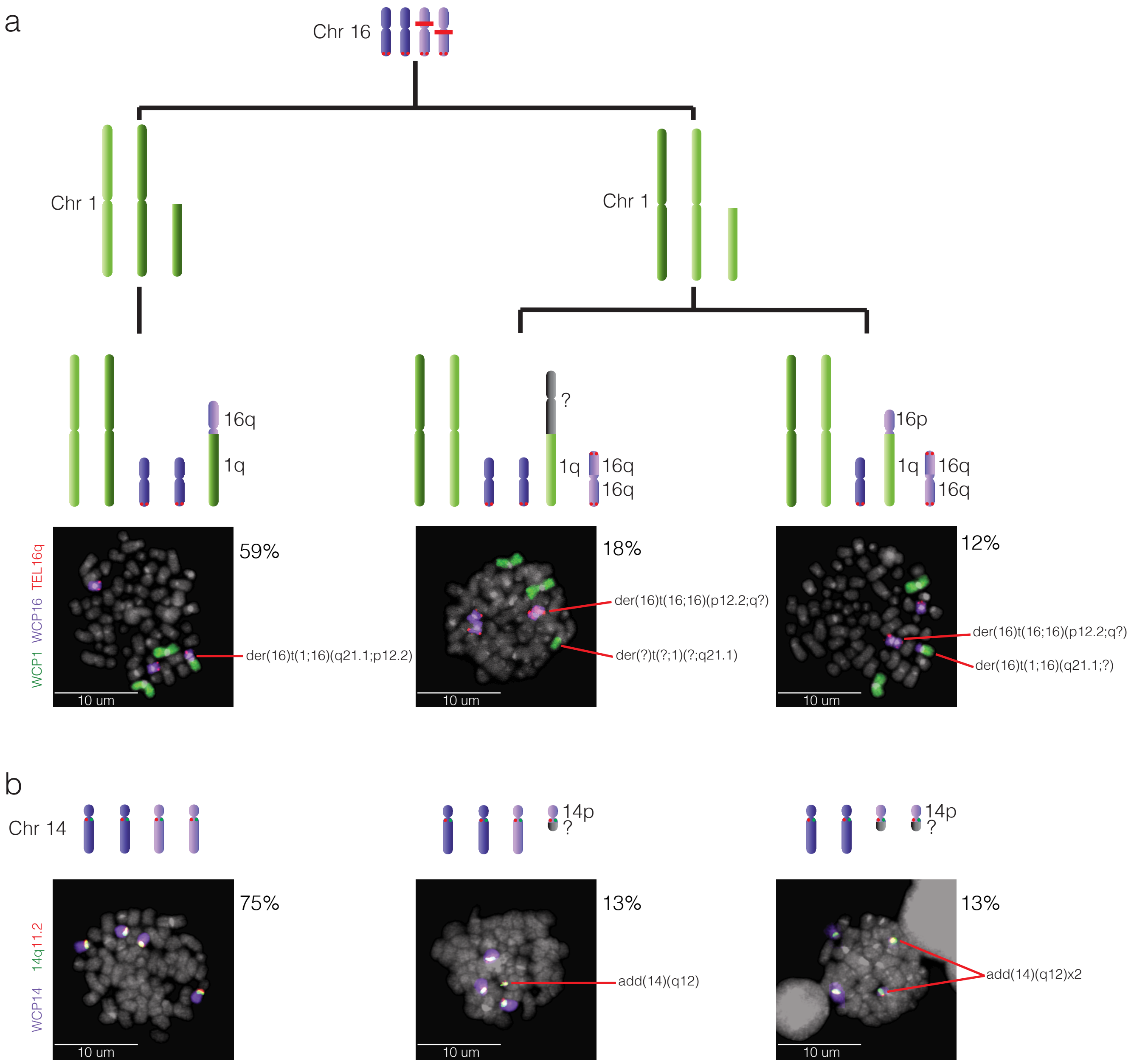

Supplementary Figure 4. Complex patterns of copy number changes involving structural rearrangements in high hyperdiploid ALL cases as elucidated by fluorescence in situ hybridization; in the schematic drawings, different shades of each color represent different homologues. Twenty to thirty-nine metaphase cells were independently analyzed for each case, with percentages of cells corresponding to each subclone shown in the figure. A) Multiple rearrangements involving chromosome 16 in case 3. A traumatic event likely caused breakage in two different chromosomes 16 (at 16p12.2 and 16q) in a cell with tetrasomy 16 as well as two separate gains of 1q, resulting in three subclones with different chromosome 1 and 16 rearrangements. B) Chromosome 14 rearrangements in case 4. Two events of loss of 14q resulted in three subclones with a tetrasomy 14, three normal copies of chromosome 14, and an add(14)(q12), and two normal copies of chromosome 14 and two identical add(14)(q12), respectively. Abbreviations: TEL16q, telomere 16q; WCP, whole chromosome paint. Schematics created with BioRender.com.

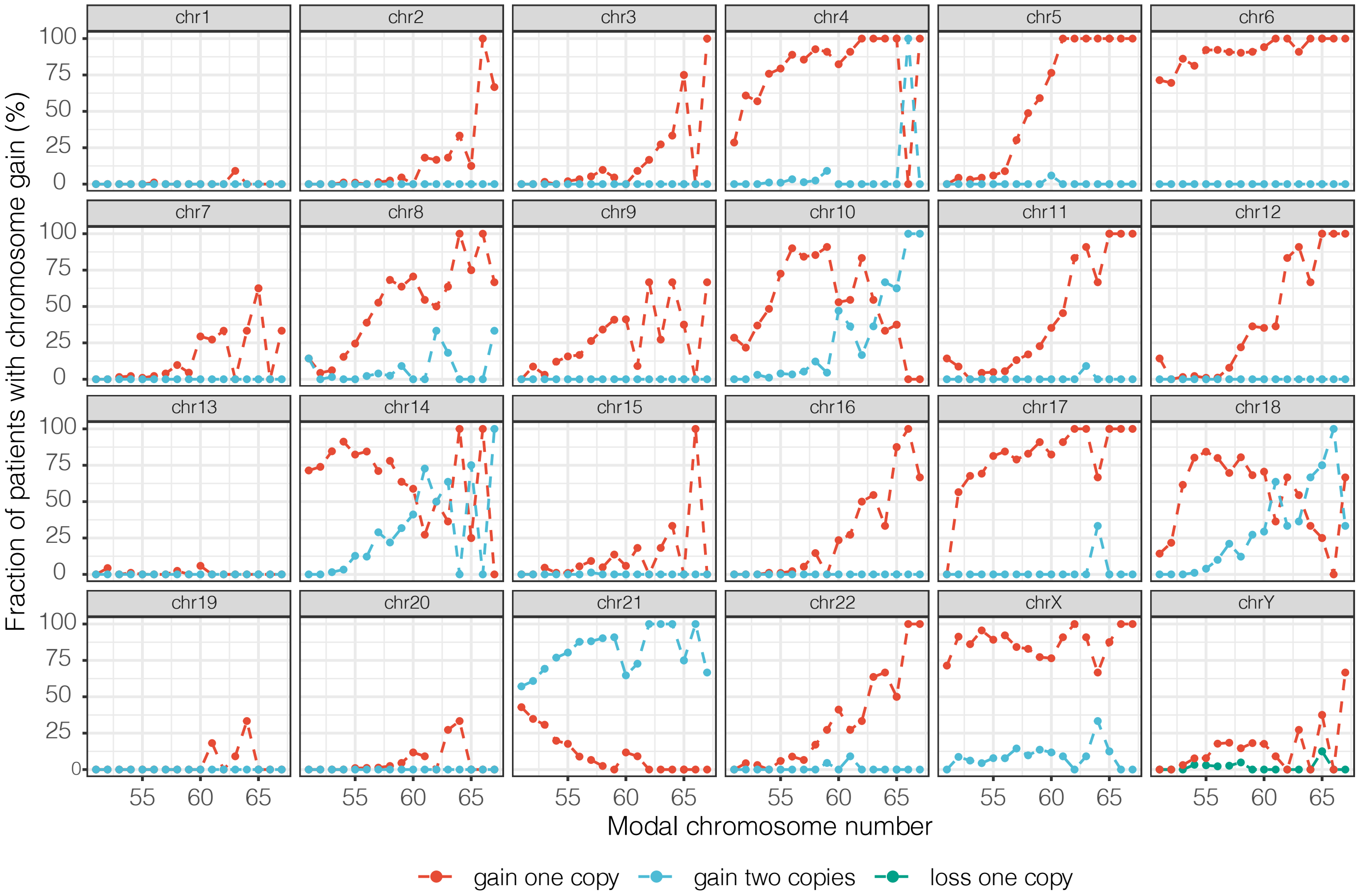

Supplementary Figure 5. Gain of each chromosome per modal number. Source data are provided as a Source Data file.

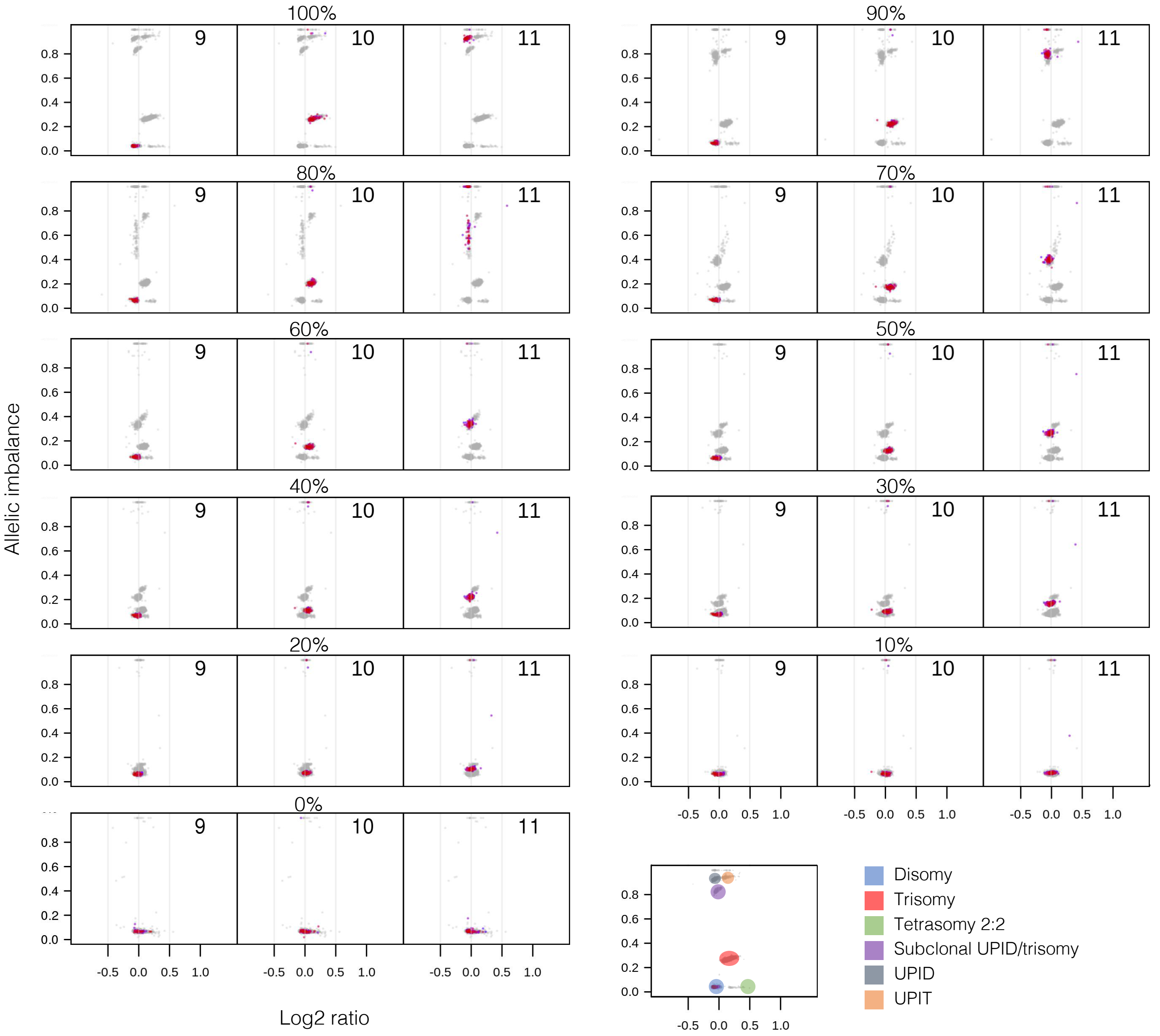

Supplementary Figure 6. SNP array analysis of a dilution series with 0-100% leukemic cells. The series was made using a leukemic sample with close to 100% leukemic blast cells and its corresponding remission sample, with 0% blast cells. Results are from TAPS[1] and graphs show the allelic imbalance versus the log2 ratio. Signals from chromosomes 9 (disomic), 10 (trisomic) and 11 (uniparental isodisomy; UPID) are shown in red. The legend shows where the signal from chromosomes with a specific copy number clusters in the pure leukemic sample. UPID11 can be detected at 20% and trisomy 10 at 30% leukemic cells, corresponding to these clone sizes. The dilution series SNP array data have previously been published in Paulsson et al.[2] Abbreviations: UPID, uniparental isodisomy; UPIT, uniparental isotrisomy.

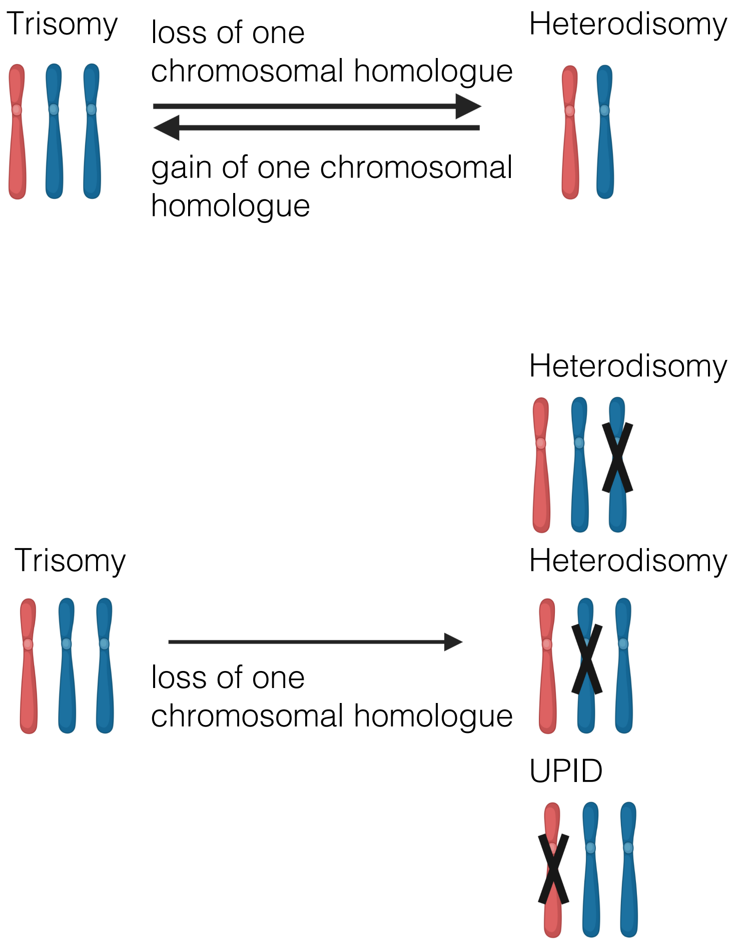

Supplementary Figure 7. Schematics of how subclonal populations may arise. The top panel shows how heterodisomy/trisomy could arise either by an initial disomy becoming a trisomy or vice versa, i.e. the direction of the change cannot be inferred. The bottom panel shows that uniparental isodisomy(UPID)/trisomy can only arise from initial trisomy by loss of one chromosomal homologue. Here, 2/3 cells become heterodisomies and 1/3 cells becomes a UPID. Abbreviations: UPID, uniparental isodisomy. Created with BioRender.com.

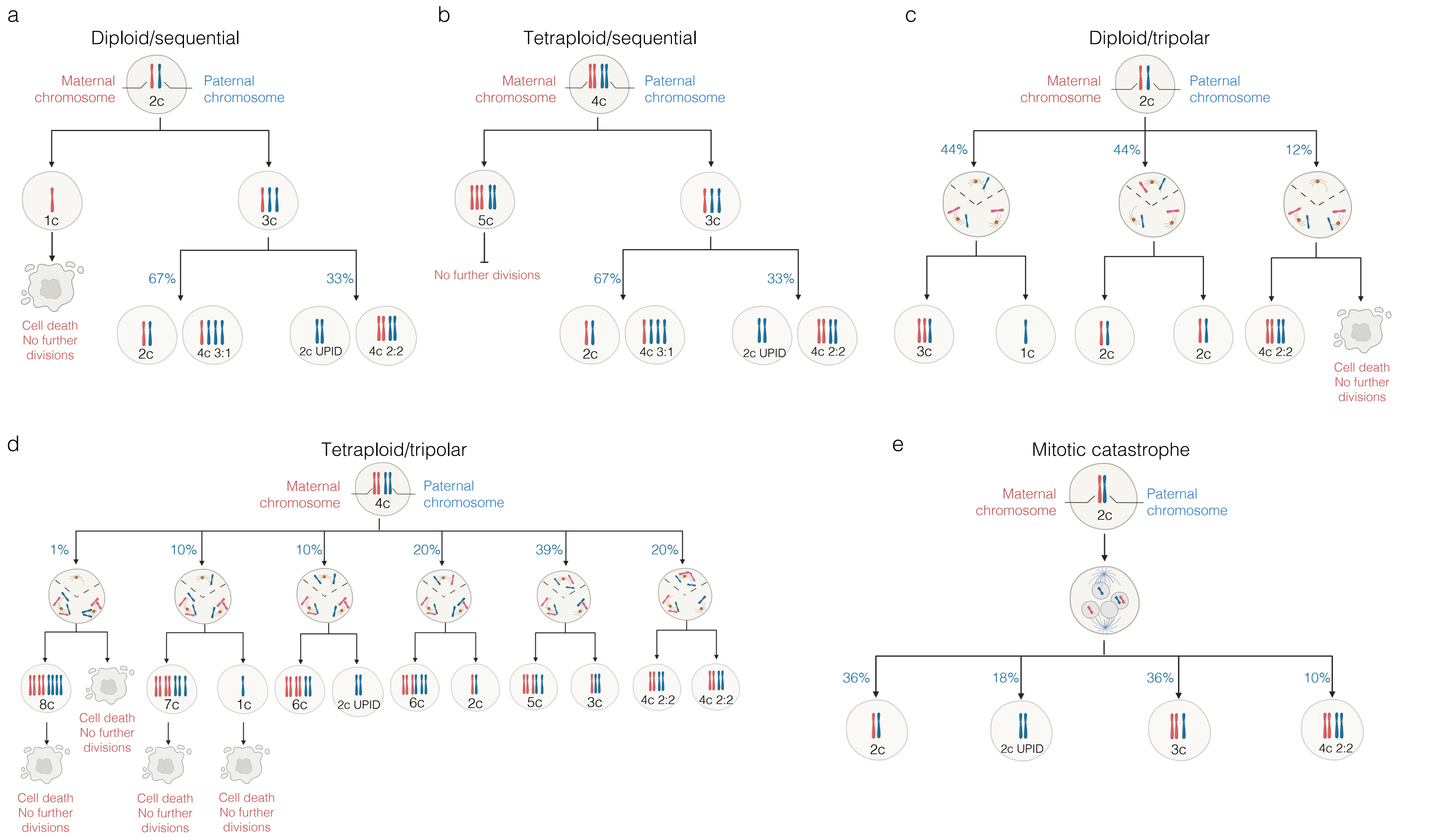

Supplementary Figure 8. Schematics of how five different models for hyperdiploidy development will lead to different patterns of tetrasomies 2:2 and 3:1 and uniparental isodisomies. Abbreviations: c, copy/copies; UPID, uniparental isodisomy. Created with BioRender.com.

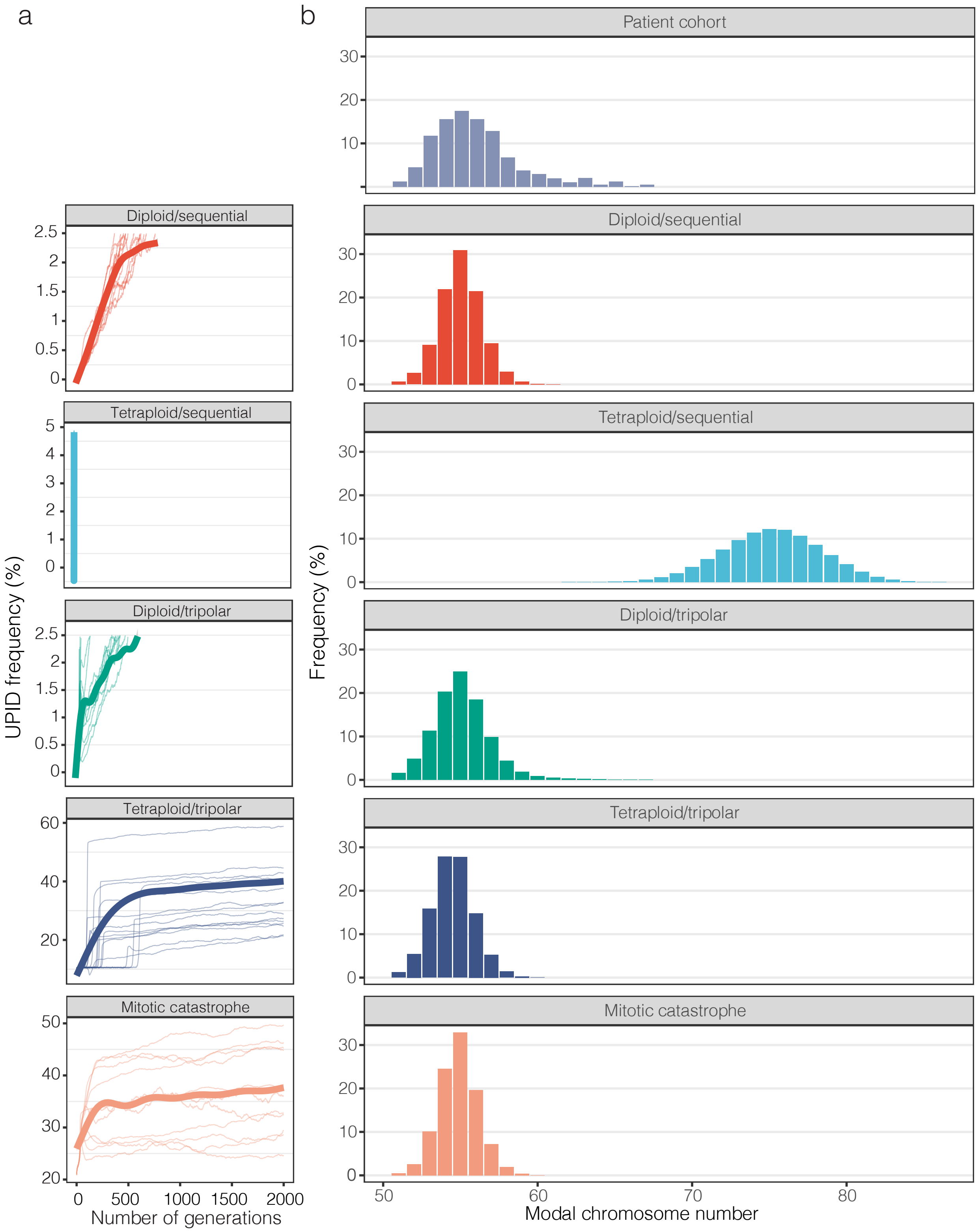

Supplementary Figure 9. Uniparental isodisomy (UPID) frequencies and distribution of modal chromosome numbers (MCN). A. UPID frequencies for groups weak pos and neg chromosomes over 2,000 generations in each simulation model. Thick lines show the generalized additive model regression of UPID frequencies of all simulations and thin lines show the UPID frequencies of 15 randomly selected simulations. The diploid/tripolar and diploid/sequential models reached 2.5% UPIDs after 50-800 generations, consistent with the patient data. B. Distribution of MCN in the patient cohort and in the five simulation models. All simulation models but the tetraploid/sequential model resulted in a similar MCN distribution to the patient cohort. The tetraploid/sequential model resulted in very few cells that had MCN 51-67; most of the virtual cells showed MCN around 75. Abbreviations: UPID, uniparental isodisomy. Source data are provided as a Source Data file.

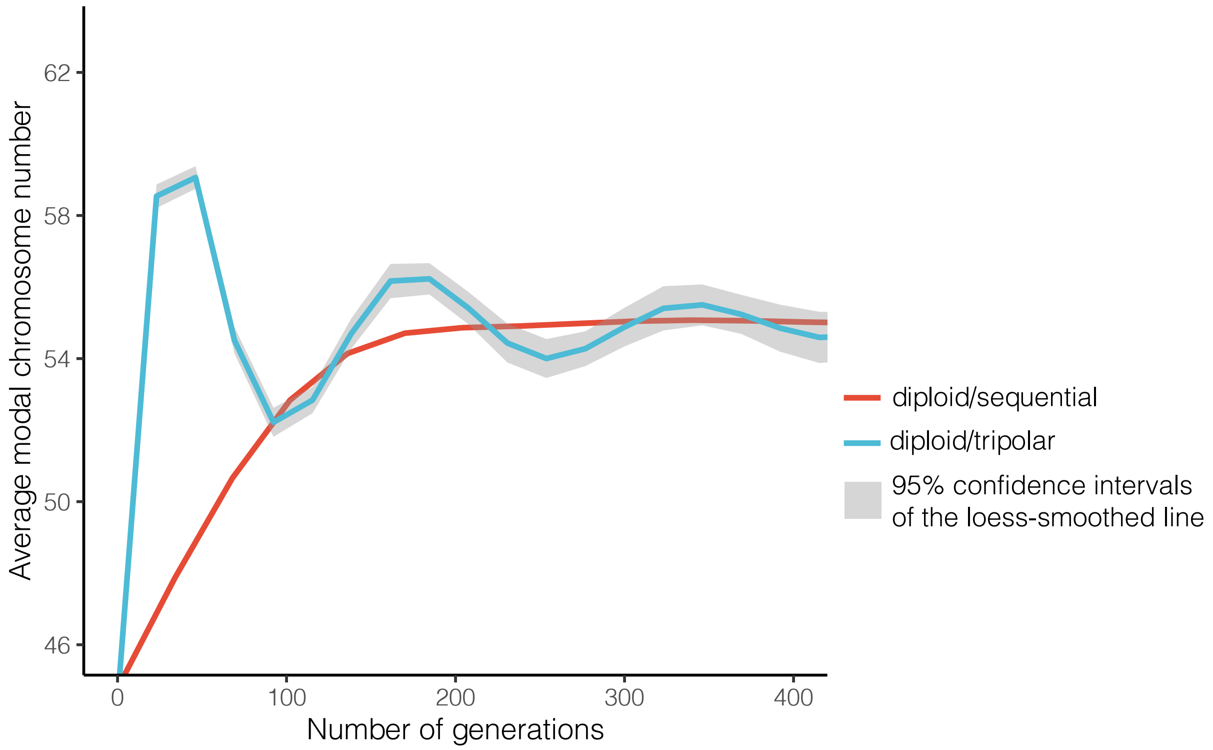

Supplementary Figure 10. Models of chromosome copy number evolution during high hyperdiploidy development. The LOESS-smoothed lines show the correlation between the average modal chromosome number (Y axis) and the number of simulated generations (X-axis) of 100 randomly sampled simulation results from the diploid/sequential model and diploid/tripolar model. The gray ribbon shows the 95% confidence intervals of the loess-smoothed line. An initial burst of whole chromosome gain events was observed in the diploid/tripolar model. These events were followed by a period of transient instability and stable expansions during the high hyperdiploidy development, in line with the punctuated copy number evolution model. In the diploid/sequential model, chromosomes were acquired sequentially throughout high hyperdiploidy development, indicating gradual copy number evolution. Abbreviations: LOESS, locally weighted scatterplot smoothing. Source data are provided as a Source Data file.

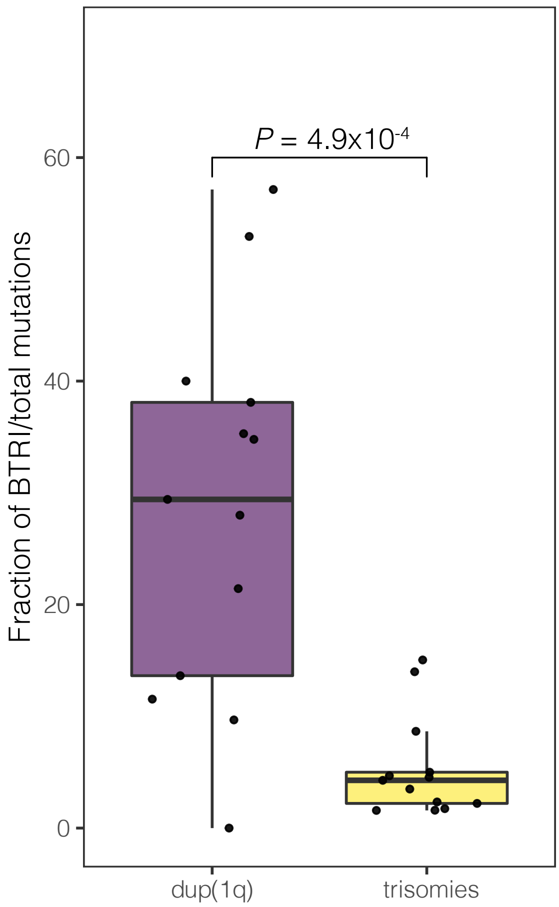

Supplementary Figure 11. Fraction of BTRI mutations (occurring before the shift to three copies) in dup(1q) compared with trisomies in the same cases. The fraction of BTRI mutations is significantly higher in dup(1q) (P = 4.9x10^{-4}; Mann-Whitney two-sided test), indicating that this rearrangement is formed subsequently to the trisomies. The centre of the boxplot is the median and lower/upper hinges correspond to the first/third quartiles; whiskers are 1.5 times the interquartile range and data beyond this range are plotted as individual points. Source data are provided as a Source Data file.

References

1. Rasmussen, M., Sundström, M., Göransson Kultima, H., Botling, J., Micke, P., Birgisson, H., Glimelius, B. & Isaksson, A. Allele-specific copy number analysis of tumor samples with aneuploidy and tumor heterogeneity. *Genome Biol* **12**, R108 (2011).
2. Paulsson, K., Lilljebjörn, H., Biloglav, A., Olsson, L., Rissler, M., Castor, A., Barbany, G., Fogelstrand, L., Nordgren, A., Sjögren, H., Fioretos, T. & Johansson, B. The genomic landscape of high hyperdiploid childhood acute lymphoblastic leukemia. *Nat Genet* **47**, 672-676 (2015).
