# Supplementary material for: Clonal origin and development of high hyperdiploidy in childhood acute lymphoblastic leukaemia
Source: Nat Commun. 2023 Mar 25;14:1658. doi: 10.1038/s41467-023-37356-5 (PMC10039905; doi:10.1038/s41467-023-37356-5)
Supplement: Supplementary file 3 — Description of Additional Supplementary File [file 41467_2023_37356_MOESM3_ESM.pdf]

Description of Addition Supplementary Information Files

Supplementary Data 1. Chromosomal patterns in 577 high hyperdiploid pediatric acute lymphoblastic leukemias.

Supplementary Data 2. Copy number comparison between samples obtained at diagnosis and at relapse.

Supplementary Data 3. Driver single nucleotide variants identified in 216 cases of high hyperdiploid pediatric acute lymphoblastic leukemia

Supplementary code - HeH_simulation is a framework for modeling high hyperdiploid childhood acute lymphoblastic leukemia cell expansion. It uses five different models to determine how a cell may become hyperdiploid.
