# Supplementary material for: Clonal origin and development of high hyperdiploidy in childhood acute lymphoblastic leukaemia
Source: Nat Commun. 2023 Mar 25;14:1658. doi: 10.1038/s41467-023-37356-5 (PMC10039905; doi:10.1038/s41467-023-37356-5)
Supplement: Supplementary file 5 — Supplementary code [file 41467_2023_37356_MOESM5_ESM.zip › HeH_simulation-main/README.pdf]

Description

HeH_simulation is a framework for modeling high hyperdiploid childhood acute lymphoblastic leukemia cell expansion. It uses five different models to determine how a cell can become hyperdiploid.
We included five possible routes to aneuploidy that have been reported to occur in cancer (https://pubmed.ncbi.nlm.nih.gov/19546858/):

1. Sequential gains in a diploid cell (diploid/sequential),
2. Initial tetraploidy followed by chromosomal losses (tetraploid/sequential),
3. Tripolar division in a diploid cell (diploid/tripolar),
4. Tripolar division in a tetraploid cell (tetraploid/tripolar),
5. Mitotic catastrophe resulting from complete loss of sister chromatid cohesion (mitotic catastrophe).

For simplicity, we only work with human genomes. All scenarios started with 46,XX cells and the Y chromosome was not included in the analysis.

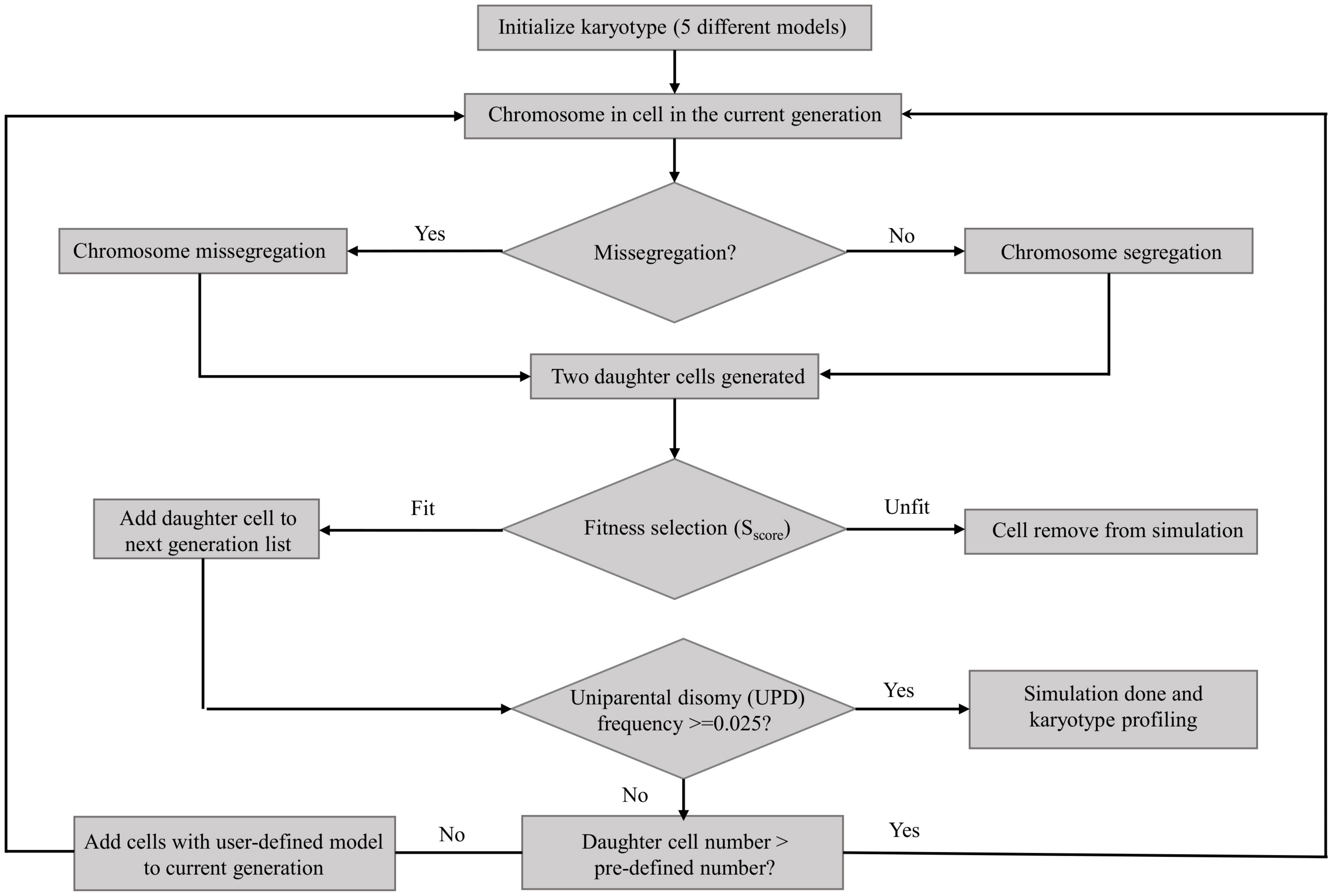

Dependency

The current implementation of HeH_simulation is tested on CentOS 7 with Python 2.7.15 and the following Python packages:
Miniconda or Bioconda
numpy=1.16.6
scipy=1.2.1

If you choose to install with Miniconda, please first follow the bioconda install procedure from here:
https://docs.conda.io/en/latest/miniconda.html#installing
If you choose to install with Bioconda, please first follow the bioconda install procedure from here:
https://bioconda.github.io/user/install.html

Installation

```
$ git clone https://github.com/yang-mj/HeH_simulation.git
or
simply unpacking the source to a working directory
```

Prerequisites

After cloning this repository to a folder of your choice, it is recommended to create a conda environment with the accompanying `HeHsimu.yaml`. In the main folder of the cloned repository, execute the following command:

```
$ conda env create -f HeHsimu.yaml
```

This will create a conda environment HeHsimu containing all dependencies for HeH_simulation.

Usage

Before you start running the python script HeH_simulation_wrapper.py, please activate the HeHsimu environment:

```
$ conda activate HeHsimu
```

To start the main pipeline, execute the following command:

```
$ python HeH_simulation_wrapper.py [options]
```

Options:

```
 -h, --help              show this help message and exit
 -i, --pinput <file>     input pre-defined virtual cells matrix (optional)
 -c, --chrgroup <int>    pre-defined number of chromosome groups, 3 or 4 (required)
 -m, --mode <int>        simulation model (required), should be one of following model (required)
                          1 : tripolar division in a diploid cell (diploid/tripolar)
                          2 : initial tetraploidy followed by chromosomal losses (tetraploid/sequential)
                          3 : sequential gains in diploid cell (diploid/sequential)
                          4 : mitotic catastrophe resulting from complete loss of sister chromatid cohesion (mitotic
catastrophe)
                          5 : tripolar division in a tetraploid cell (tetraploid/tripolar)

 -g, --generation <int>  maximum number of generation (required)
 -n, --number <int>      number of virtual cell for simulation (required)
 -t, --thread <int>      number of threads (required)
```

Run HeH_simulation with automatically generated virtual cells matrices :

```
$ python HeH_simulation_wrapper.py --chrgroup 3 --mode 1 --generation 2000 --number 50000 --thread 20 > upid.log
```

Run HeH_simulation with pre-defined virtual cells matrix:

```
$ python HeH_simulation_wrapper.py --pinput data/cell_matrix.txt --chrgroup 3 --mode 1 --generation 2000 --number 50000
--thread 20 > upid.log
```

Advanced configuration and parameters

parameter_settings.py

The parameter_settings.py provides the best-fit parameters for modeling high hyperdiploid childhood acute lymphoblastic leukemia cell expansion. These parameters need to be optimized to adapt to other projects

1). The parameter `aneuploidy_penalty_factor` is used only to model sequential gains in a diploid cell (diploid/sequential), tripolar division in a diploid cell (diploid/tripolar), tripolar division in a tetraploid cell (tetraploid/tripolar), and mitotic catastrophe resulting from complete loss of sister chromatid cohesion (mitotic catastrophe)
To model initial tetraploidy followed by chromosomal losses (tetraploid/sequential), `aneuploidy_penalty_factor` was set to 0.

2). The parameter `tetra_loss_rate` is used only to model initial tetraploidy followed by chromosomal losses (tetraploid/sequential)

chromosome_group_all.py

The program can divide all chromosomes into either three groups or four groups. Users can change the integer value in NumPy arrays g3 and g4 by providing a list of 23 integer values representing the group of chromosome 1-22, X. Currently the program can accept integer values 1, 2, 3 for the three chromosome groups model or 1, 2, 3, 4 for the four chromosome groups model.
In the program, chromosomes assigned to integer values 1, 3 or 4 is subjected to positive selection for chromosome gain whereas chromosomes assigned to integer value 2 are subjected to negative selection for chromosome gain.
Users can define the strength of selection pressures by providing a non-negative numeric value to parameter `prolification_factor_A`, `prolification_factor_B`, `prolification_factor_C` and `prolification_factor_D` for chromosomes that are assigned to group 1, 2, 3 and 4, respectively, in file `parameter_settings.py`.

Inputs --pinput (optional)

cell_matrix.txt

A tab-delimited text file contains the karyotype of each virtual cell at the beginning of the simulation. Each row of data contains the chromosome copy number of one virtual cell, ordered from chromosome 1–22, X. The chromosome copy number was defined by an integer value.
The data/cell_matrix.txt file provided by the program contains 20,000 virtual cells that were generated by the diploid/tripolar model.

Abbreviations of chromosome copy number

0 : Nullisomy
1 : Monosomy
2 : Disomy
-2 : Uniparental isodisomy (UPID; disomies involving two copies of the same chromosomal homologue)
3 : Trisomy

4 : Tetrasomy generated by a triplication of one homologue
-4 : Tetrasomy generated by duplication of both homologues
5 : Pentasomy generated by a triplication of one homologue and duplication of the other homologue
-5 : Pentasomy generated by a tetraplication of one homologue

Outputs

chr_dis file

This file contains the number of virtual cells under a particular chromosomal copy number for chromosomes 1-22,X for each generation during the simulation. The first column refers to the chromosome copy number and the following columns show the number of virtual cells for each chromosome (1-22, X). The generation numbers are shown with a "#generation_" at the beginning of line.

Abbreviations of chromosome copy number

0 : Nullisomy
1 : Monosomy
2 : Disomy
-2 : Uniparental isodisomy (UPID; disomies involving two copies of the same chromosomal homologue)
3 : Trisomy
4 : Tetrasomy generated by a triplication of one homologue
-4 : Tetrasomy generated by duplication of both homologues
5 : Pentasomy generated by a triplication of one homologue and duplication of the other homologue
-5 : Pentasomy generated by a tetraplication of one homologue

upid.log file

This file contains the running information, including

1. The generation number,
2. Number of living cell at the end of each generation,
3. Uniparental isodisomy (UPID; disomies involving two copies of the same chromosomal homologue) frequency at the end of each generation,
4. Running time for each generation during the simulation.

i_simulation.txt file

This file contains the karyotype of each virtual cell at the beginning of the simulation. Each row of data contains the chromosome copy number of one virtual cell, ordered from chromosome 1–22, X. The chromosome copy number was defined by an integer value.

simulation.txt file

This file contains the karyotype of each virtual cell at the end of the simulation. Each row of data contains the chromosome copy number of one virtual cell, ordered from chromosome 1–22, X. The chromosome copy number was defined by an integer value.

Abbreviations of chromosome copy number

0 : Nullisomy
1 : Monosomy
2 : Disomy
-2 : Uniparental isodisomy (UPID; disomies involving two copies of the same chromosomal homologue)
3 : Trisomy
4 : Tetrasomy generated by a triplication of one homologue
-4 : Tetrasomy generated by duplication of both homologues
5 : Pentasomy generated by a triplication of one homologue and duplication of the other homologue
-5 : Pentasomy generated by a tetraplication of one homologue

About this repository

The repository was created to illustrate how the hyperdiploidy in high hyperdiploid childhood acute lymphoblastic leukemia originates. This is discussed in detail in this manuscript:
Clonal origin and development of high hyperdiploidy in childhood acute lymphoblastic leukemia
