# Supplementary figures and images for: Clonal origin and development of high hyperdiploidy in childhood acute lymphoblastic leukaemia
Source: Nat Commun. 2023 Mar 25;14:1658. doi: 10.1038/s41467-023-37356-5 (PMC10039905; doi:10.1038/s41467-023-37356-5)

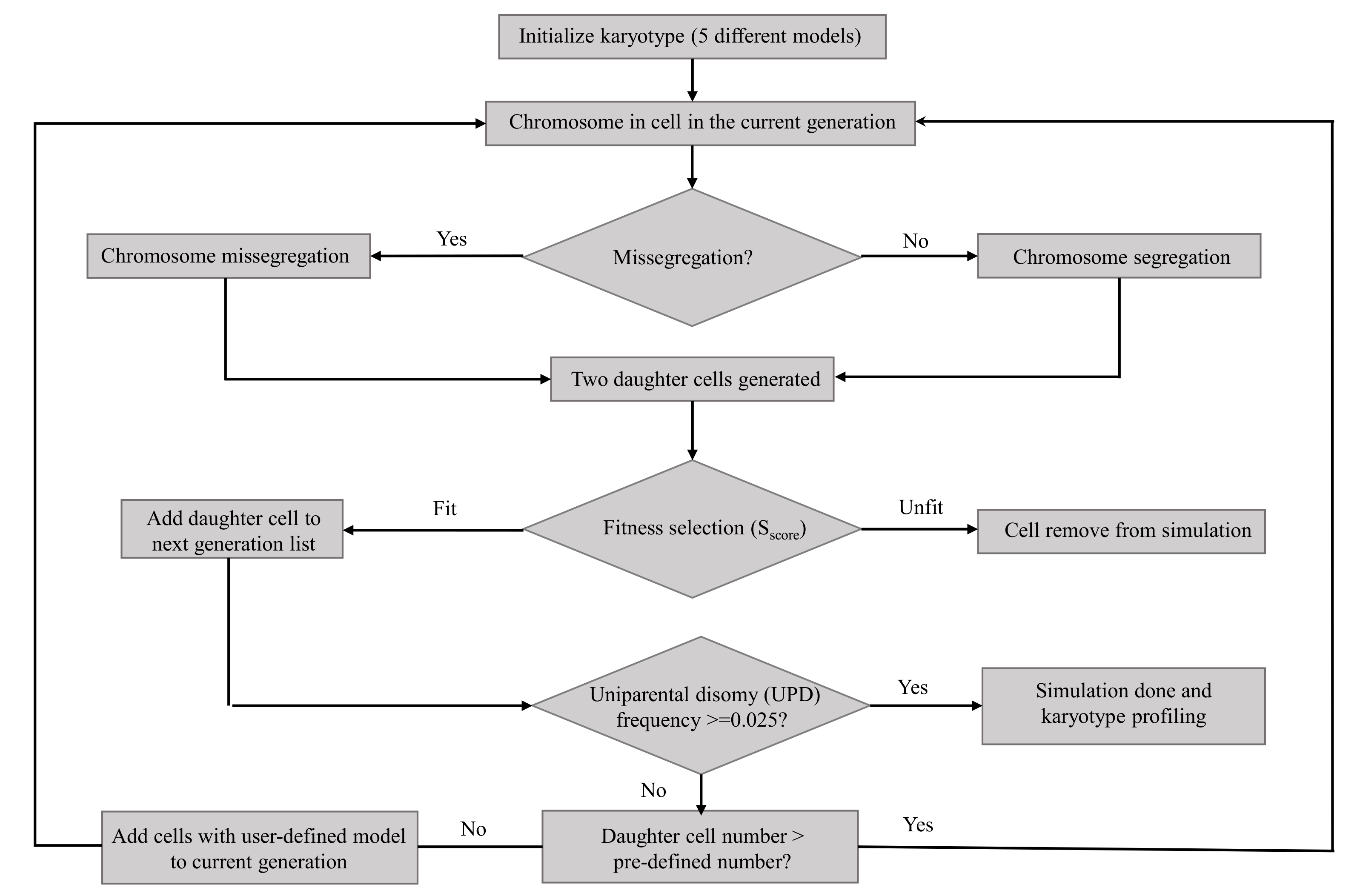

Supplement: Supplementary file 5 — Supplementary code [file 41467_2023_37356_MOESM5_ESM.zip › HeH_simulation-main/docs/imgs/HeH_simulation_flowchart.png]
